# An intron SNP rs2069837 in *IL-6* is associated with osteonecrosis of the femoral head development

**DOI:** 10.1186/s12920-021-01142-3

**Published:** 2022-01-05

**Authors:** Ruisong Wang, Rui Li, Ruiyu Liu

**Affiliations:** 1 Department of Orthopedics, the Second Affiliated Hospital of Xi’an Jiaotong University, Xi’an, 710004 China; 2 Department of orthopedics, Xi’an Fifth Hospital, Xi’an, 710082 China; 3 Department of rheumatology, Xi’an Fifth Hospital, Xi’an, 710082 China

**Keywords:** ONFH, *IL-6*, Susceptibility, Polymorphism

## Abstract

**Background:**

Genetic polymorphisms play a crucial role in the development of osteonecrosis of the femoral head (ONFH). This study mainly explored the association of *IL-6* variants and ONFH susceptibility among the Chinese Han population.

**Methods:**

Two variants (rs2069837, and rs13306435) in the *IL-6* gene were identified and genotyped from 566 patients with ONFH and 566 healthy controls. The associations between *IL-6* polymorphisms and ONFH susceptibility were assessed using odds ratio (OR) and 95% confidence interval (95% CI) via logistic regression. The potential function of these two variants was predicted by the HaploReg online database.

**Results:**

The results of the overall analysis revealed that *IL-6* rs2069837 was correlated with decreased risk of ONFH among the Chinese Han population (*p* < 0.05). In stratified analysis, rs2069837 also reduced the susceptibility to ONFH in older people (> 51 years), males, nonsmokers, and nondrinkers (*p* < 0.05). However, no associations between rs13306435 and ONFH susceptibility were observed (*p* > 0.05).

**Conclusions:**

To sum up, we suggested that rs2069837 G>A polymorphism in the *IL-6* gene was significantly associated with a decreased risk of ONFH among the Chinese Hans. These findings underscored the crucial role of *IL-6* rs2069837 in the occurrence of ONFH.

**Supplementary Information:**

The online version contains supplementary material available at 10.1186/s12920-021-01142-3.

## Background

Osteonecrosis of the femoral head (ONFH) refers to the death of some osteocytes or the necrosis of some marrow elements caused by venous congestion, impairment or interruption of arterial blood, and subsequent repair, which in turn causes the necrosis of bone tissues [1]. The number of ONFH patients is increasing worldwide year by year [2]. The incidence of ONFH was 2.91 cases per 100,000 person-years in the Japanese population [3]. In Korea, the estimated yearly prevalence increased from 20.53/100,000 in 2002 to 37.96/100,000 in 2006, and the average number of new cases annually has been estimated at 14,103 [4]. Based on Chinese population data from 2010, there are approximately 8.12 million cases of ONFH among Chinese people aged 15 years and over [5]. And it poses a severe financial burden for both individuals and healthcare systems. However, the pathophysiology of ONFH has not been elucidated.

There is a wide spectrum of aetiological risk factors in ONFH including alcohol use, glucocorticoid use, and genetic factors [6, 7]. Up to now, the role of genetic polymorphisms in the development of ONFH has been widely reported. For example, Zhao et al. found that NOS3 variants were associated with the occurrence of ONFH [8]. A meta-analysis by Song et al. showed that eNOS polymorphism was correlated with idiopathic and secondary ONFH in Caucasians and Asians [9]. In addition, increasing studies have documented that candidate gene polymorphisms (e.g. *RANK*, *OPG*, *RTEN*, TNF-α) can affect the susceptibility to ONFH [10–12].

Interleukin-6 (*IL-6*) is a major pro-inflammatory cytokine that participants in the pathophysiological process of many diseases [13, 14]. Meanwhile, the function of IL-6 in bone disease has been extensively studied. Ding et al. reported that IL-6 was decreased in older adults, and it could serve as a predictor of bone loss and resorption [15]. IL-6 stimulated osteoclastogenesis by increasing RANKL gene expression by osteoblasts [16]. Yamaguchi et al. have shown that ischemic osteonecrosis may increased IL-6 levels in the synovial fluid [17]. In addition, IL-6 was found to be involved in the pathogenesis of rheumatoid arthritis and osteoarthritis [18, 19]. These findings suggest that IL-6 played a crucial role in the occurrence of bone disease. The association of *IL-6* rs2069837, and rs13306435 polymorphisms with the risk of rheumatoid arthritis and lumbar disc disease were assessed [20, 21], but not studies on ONFH.

In the present study, we aimed to investigate the effect of *IL-6* rs2069837 and rs13306435 polymorphisms on ONFH susceptibility among Chinese Hans.

## Materials and methods

### Study subjects

In this case–control study, we recruited 566 patients with ONFH and 566 healthy controls. ONFH patients were diagnosed by examining osteonecrosis in anteroposterior and frog view X-rays of both hips and/or magnetic resonance imaging. All patients were selected randomly from The Second Affiliated Hospital of Xi’an Jiaotong University. The patients without other direct trauma, cardiovascular diseases, rheumatoid arthritis, ankylosing spondylitis, hip joint-involving diseases (like hip dysplasia), diabetes mellitus, renal dysfunction, cancer, corticosteroids, alcohol use, and familial hereditary diseases were included. The healthy control group enrolled from the same hospital during the same period. The healthy controls were included if individuals met the following criteria: (1) No hip pain; (2) Anteroposterior and frog-leg lateral pelvic radiographs did not show any lesions; (3) Subjects without a long term of alcohol use and steroid use.

This study was approved by the Ethics Committee of the hospital and followed the Declaration of Helsinki. The informed consent of all subjects was obtained before the experiment.

### SNP selection and genotyping

Based on previous studies [20, 21], 1000 Genomes Chinese Han Beijing population and dbSNP database (https://www.ncbi.nlm.nih.gov/snp/) with a minor allele frequency (MAF) > 0.01, and Hardy–Weinberg equilibrium (HWE) > 0.05, we selected rs2069837 and rs13306435 in *IL-6* gene for genotyping. Genomic DNA was extracted from peripheral blood samples using the GoldMag DNA Extraction Kit (GoldMag Co. Ltd, Xi’an, China). The concentration and purity of DNA were assessed using the NanDrop 2000 (Thermo Scientific, USA).

The primer sequence of rs2069837 and rs13306435 was presented in Additional file 1: Table S1. PCR reactions were performed in a buffer containing 1 μl DNA, 0.5 μl PCR Buffer, 0.4 μl MgCl_2_, 0.1 μl dNTP Mix, 1.0 μl primer mix, and 0.2 μl Taq ligase in a final reaction volume of 5 μl. The reaction mixture was heated to 94 °C for 15 min for denaturation. Then, the sample was subjected to 45 cycles of 94 °C 20 s, annealing at 56 °C 30 s and extension at 72 °C 60 s, followed by a final extension step at 72 °C for 3 min. The PCR product was used to genotype using the Agena MassArray platform (Agena Bioscience, San Diego, CA, USA) [21, 22]. Then, the raw data was analyzed and managed using the Agena Typer 4.0 software.

### Statistical analysis

The demographic characteristics (age and sex) were assessed in the case and control groups using the student *t*-test and χ^2^ test. Hardy–Weinberg equilibrium (HWE) of each SNP among controls was evaluated using the χ^2^ test. The correlation between *IL-6* polymorphisms (rs2069837, rs13306435) and ONFH susceptibility was examined using odds ratio (OR) and 95% confidence interval (CI) by logistic regression by plink 1.9 software (http://zzz.bwh.harvard.edu/plink/). The functional annotation of each SNP was predicted by the HaploReg v4.1 database (https://pubs.broadinstitute.org/mammals/haploreg/haploreg.php). We used String database and Cytoscape software to generate protein–protein interaction networks. Then, Gene Ontology (GO) and Kyoto encyclopedia of genes and genomes (KEGG) on mRNAs were performed to explore their functions using Cluster Profiler in R package. The ggplot2 in R package was used to draw the GO and KEGG analysis results. A *p* value < 0.05 was considered statistically significant.

## Results

### Characteristics of study participants

In this study, we enrolled 566 patients with an average age of 51.34 ± 14.51 years and 566 healthy controls with an average age of 51.10 ± 13.93 years, respectively. There was no significant difference in terms of age (*p* = 0.503) and sex (*p* = 0.858) between the case and control group (Table 1). There were 223 cases of stage III/IV and 77 cases of stage I/II.

**Table 1:** Characteristics of ONFH patients and controls

Variables	Cases (N = 566)	Control (N = 566)	*p* value
Age, years	51.34 ± 14.51	51.10 ± 13.93	0.503^a^
≤ 51	307 (54.2%)	295 (52.1%)	
> 51	259 (45.8%)	271 (47.9%)	
Sex			0.858^b^
Male	314 (55.5%)	311 (55.0%)	
Female	252 (44.5%)	255 (45.0%)	
Smoking			
Yes	256 (45.2%)	300 (53.0%)	
No	310 (54.8%)	266 (47.0%)	
Drinking			
Yes	293 (51.8%)	285 (50.4%)	
No	273 (48.2%)	281 (49.6%)	
Stage			
III/IV	223 (39.0%)		
I/II	77 (14.0%)		
Missing	300 (47%)		

^a^*p* values were calculated from independent sample *t*-test

^b^*p* values were calculated from two-sided χ^2^ test

In addition, the basic information of *IL-6* polymorphisms was presented in Table 2. Rs2069837 is an intronic polymorphism, and rs13306435 is a missense polymorphism. Furthermore, these two SNPs were consistent with HWE (*p* > 0.05). The functional role of rs2069837 and rs13306435 are associated with regulation of promoter histone marks, enhancer histone marks, DNAse, proteins bound and motifs changed.

**Table 2:** Basic information of SNPs in IL-6 gene

SNP	Chr: position	Function	Allele (minor/major)	MAF in Case	MAF in Control	O (HET)	E (HET)	HWE *p*	HaploReg
rs2069837	7: 22728408	Intronic	G/A	0.183	0.227	0.356	0.351	0.905	Promoter histone marks; Enhancer histone marks; DNAse; Proteins bound; Motifs changed
rs13306435	7: 22731420	Missense	A/T	0.034	0.038	0.072	0.073	0.564	Promoter histone marks; Enhancer histone marks; DNAse; Motifs changed

*SNP* single nucleotide polymorphism, *MAF* minor allele frequency, *HWE* Hardy–Weinberg equilibrium

### Associations between rs2069837, rs13306435 and ONFH susceptibility

The results of Table 3 revealed that rs2069837 decreased the susceptibility to ONFH in the allele (OR = 0.76, 95% CI = 0.62–0.93, *p* = 0.009), heterozygote (OR = 0.75, 95% CI = 0.58–0.96, *p* = 0.023), dominant (OR = 0.73, 95% CI = 0.57–0.93, *p* = 0.011), and additive (OR = 0.76, 95% CI = 0.62–0.93, *p* = 0.009) models. In stratified analysis (Table 4), rs2069837 was also correlated with a lower-risk of ONFH in males, non-smokers and individuals aged > 51 years old in the allele, heterozygote, dominant, and additive models (*p* < 0.05). Additionally, rs2069837 only reduced the risk of ONFH in non-drinkers in the heterozygote and dominant models (*p* < 0.05). However, the associations between rs13306435 and ONFH susceptibility were not observed in the overall analysis and stratified analysis (*p* > 0.05, data no shown). Besides, we also evaluated the associations between *IL-6* polymorphisms and ONFH susceptibility stratified by stage. However, no significant association was found (Additional file 1: Table S2).

**Table 3:** Associations between IL-6 polymorphisms and ONFH susceptibility

SNP	Model	Genotype	Control	Case	OR (95% CI)	*p*
rs2069837	Allele	A	873 (77.3%)	925 (81.7%)	1.00	
		G	257 (22.7%)	207 (18.3%)	0.76 (0.62–0.93)	**0.009**
	Codominant	AA	336 (59.5%)	378 (66.8%)	1.00	
		GG	28 (5%)	19 (3.4%)	0.61 (0.33–1.11)	0.103
		AG	201 (35.6%)	169 (29.9%)	0.75 (0.58–0.96)	**0.023**
	Dominant	AA	336 (59.5%)	378 (66.8%)	1.00	
		GG + AG	229 (40.5%)	188 (33.2%)	0.73 (0.57–0.93)	**0.011**
	Recessive	AA + AG	537 (95%)	547 (96.6%)	1.00	
		GG	28 (5%)	19 (3.4%)	0.67 (0.37–1.21)	0.186
	Additive	/			0.76 (0.62–0.93)	**0.009**
rs13306435	Allele	T	1089 (96.2%)	1094 (96.6%)	1.00	
		A	43 (3.8%)	38 (3.4%)	0.88 (0.56–1.37)	0.572
	Codominant	TT	524 (92.6%)	528 (93.3%)	1.00	
		AA	1 (0.2%)	0 (0%)	/	/
		AT	41 (7.2%)	38 (6.7%)	0.92 (0.58–1.45)	0.712
	Dominant	TT	524 (92.6%)	528 (93.3%)	1.00	
		AA + AT	42 (7.4%)	38 (6.7%)	0.90 (0.57–1.41)	0.634
	Recessive	TT + AT	565 (99.8%)	566 (100%)	1.00	
		AA	1 (0.2%)	0 (0%)	/	/
	Additive	/			0.88 (0.56–1.37)	0.560

Bold indicates statistical signifcance (*p* < 0.05)

*SNP* single nucleotide polymorphism, *OR* odds ratio, *CI* confidence interval

*p* values were calculated by logistic regression analysis adjusted age and sex

**Table 4:** Relationship between IL-rs2069837 and ONFH susceptibility in different subgroups

SNP	Model	Genotype	Male				Female			
			Case	Control	OR (95% CI)	*p*	Case	Control	OR (95% CI)	*p*
Sex										
rs2069837	Allele	A	514	475	1.00		411	398	1.00	
		G	114	145	0.73 (0.55–0.96)	**0.023**	93	112	0.80 (0.59–1.09)	0.164
	Codominant	AA	210	115	1.00		168	156	1.00	
		GG	10	15	0.57 (0.25–1.31)	0.188	9	13	0.65 (0.27–1.55)	0.329
		AG	94	180	0.70 (0.50–0.98)	**0.037**	75	86	0.81 (0.56–1.19)	0.279
	Dominant	AA	210	180	1.00		168	156	1.00	
		GG + AG	104	130	0.68 (0.49–0.95)	**0.022**	84	99	0.79 (0.55–1.14)	0.203
	Recessive	AA + AG	304	295	1.00		243	242	1.00	
		GG	10	15	0.65 (0.29–1.47)	0.300	9	13	0.69 (0.29–1.65)	0.407
	Additive	/	/	/	0.72 (0.54–0.95)	**0.022**	/	/	0.81 (0.59–1.10)	0.173

SNP	Model	Genotype	> 51				≤ 51			
			Case	Control	OR (95% CI)	*p*	Case	Control	OR (95% CI)	*p*
Age										
rs2069837	Allele	A	506	449	1.00		419	424	1.00	
		G	108	139	0.69 (0.52–0.91)	**0.009**	99	118	0.85 (0.63–1.15)	0.284
	Codominant	AA	206	169	1.00		175	167	1.00	
		GG	7	14	0.41 (0.16–1.05)	0.062	12	14	0.83 (0.37–1.85)	0.645
		AG	94	111	0.69 (0.49–0.98)	**0.036**	75	90	0.81 (0.56–1.17)	0.259
	Dominant	AA	206	169	1.00		172	167	1.00	
		GG + AG	101	125	0.66 (0.48–0.92)	**0.015**	87	104	0.81 (0.57–1.16)	0.244
	Recessive	AA + AG	300	280	1.00		247	257	1.00	
		GG	7	14	0.47 (0.19–1.18)	0.108	12	14	0.89 (0.40–1.960	0.770
	Additive	/	/	/	0.68 (0.51–0.90)	**0.008**	/	/	0.85 (0.64–1.14)	0.286

SNP	Model	Genotype	Smoking				Non-smoking			
			Case	Control	OR (95% CI)	*p*	Case	Control	OR (95% CI)	*p*
Smoking										
rs2069837	Allele	A	405	455	1.00		520	418	1.00	
		G	107	145	0.83 (0.6–1.10)	0.194	100	112	0.72 (0.53–0.97)	**0.029**
	Codominant	AA	161	173	1.00		217	163	1.00	
		GG	12	18	0.69 (0.32–1.48)	0.343	7	10	0.54 (0.20–1.45)	0.220
		AG	83	109	0.82 (0.57–1.17)	0.278	86	92	0.69 (0.48–0.99)	**0.046**
	Dominant	AA	161	173	1.00		217	163	1.00	
		GG + AG	95	127	0.80 (0.57–1.13)	0.205	93	102	0.68 (0.48–0.96)	**0.029**
	Recessive	AA + AG	244	282	1.00		303	255	1.00	
		GG	12	18	0.74 (0.35–1.58)	0.438	7	10	0.60 (0.23–1.62)	0.317
	Additive	/	/	/	0.83 (0.62–1.1)	0.182	/	/	0.71 (0.52–0.96)	**0.026**

SNP	Model		Genotype		Drinking								Non-drinking							
					Case		Control		OR (95% CI)		*p*		Case		Control			OR (95% CI)		*p*
Drinking																				
rs2069837		Allele		A		476		438		1.00				449		435	1.00			
				G		110		132		0.77 (0.58–1.02)		0.067		97		125	0.75 (0.56–1.01)		0.059	
		Codominant		AA		193		169		1.00				185		167	1.00			
				GG		10		16		0.53 (0.24–1.21)		0.133		9		12	0.68 (0.28–1.67)		0.401	
				AG		90		100		0.79 (0.55–1.12)		0.182		79		101	0.69 (0.48–1.00)		**0.048**	
		Dominant		AA		193		169		1.00				185		167	1.00			
				GG + AG		100		116		0.75 (0.53–1.05)		0.098		88		113	0.69 (0.49–0.98)		**0.040**	
		Recessive		AA + AG		283		269		1.00				264		268	1.00			
				GG		10		16		0.58 (0.26–1.30)		0.187		9		12	0.77 (0.32–1.88)		0.569	
		Additive		/		/		/		0.76 (0.57–1.02)		0.063		/		/	0.74 (0.55–1.00)		0.051	

Bold indicates statistical signifcance (*p* < 0.05)

*SNP* single nucleotide polymorphism, *OR* odds ratio, *CI* confidence interval

*p* values were calculated by logistic regression analysis adjusted age and sex

### FPRP analysis

The statistical power and FPRP were calculated for all positive results. As was shown in Table 5, all of the significant findings for rs2069837 polymorphism remained noteworthy at the prior probability level of 0.25 and FPRP threshold of 0.2.

**Table 5:** False positive report probability of the association rs2069837 and ONFH susceptibility in subgroups

Model and variables	Genotype	OR (95% CI)	*p*^a^	Statistical power	Prior probability				
					0.25	0.1	0.01	0.001	0.0001
Overall analysis									
Allele	G versus A	0.76 (0.62–0.93)	0.009	0.898	0.025^b^	0.072^b^	0.459	0.896	0.988
Heterozygote	AG versus AA	0.75 (0.58–0.96)	0.023	0.825	0.075^b^	0.196^b^	0.729	0.964	0.996
Dominant	GG + AG versus AA	0.73 (0.57–0.93)	0.011	0.769	0.041^b^	0.113^b^	0.583	0.934	0.993
Additive	/	0.76 (0.62–0.93)	0.009	0.898	0.025^b^	0.072^b^	0.459	0.896	0.988
Stratification analysis									
Male									
Allele	G versus A	0.73 (0.55–0.96)	0.023	0.997	0.068^b^	0.180^b^	0.707	0.961	0.996
Heterozygote	AG versus AA	0.70 (0.50–0.98)	0.037	0.975	0.104^b^	0.258	0.793	0.975	0.997
Dominant	GG + AG versus AA	0.68 (0.49–0.95)	0.022	0.964	0.069^b^	0.182^b^	0.709	0.961	0.996
Additive	/	0.72 (0.54–0.95)	0.022	0.995	0.057^b^	0.154^b^	0.668	0.953	0.995
> 51 years									
Allele	G versus A	0.69 (0.52–0.91)	0.009	0.989	0.025^b^	0.073^b^	0.462	0.897	0.989
Heterozygote	AG versus AA	0.69 (0.49–0.98)	0.036	0.964	0.106^b^	0.263	0.797	0.975	0.997
Dominant	GG + AG versus AA	0.66 (0.48–0.92)	0.015	0.949	0.043^b^	0.119^b^	0.597	0.937	0.993
Additive	/	0.68 (0.51–0.90)	0.008	0.984	0.021^b^	0.060^b^	0.413	0.877	0.986
Non-smoking									
Allele	G versus A	0.72 (0.53–0.97)	0.029	0.992	0.085^b^	0.218	0.754	0.969	0.997
Heterozygote	AG versus AA	0.69 (0.48–0.99)	0.046	0.960	0.121^b^	0.292	0.819	0.979	0.998
Dominant	GG + AG versus AA	0.68 (0.48–0.96)	0.029	0.960	0.081^b^	0.210	0.745	0.967	0.997
Additive	/	0.71 (0.52–0.96)	0.026	0.989	0.073^b^	0.192^b^	0.723	0.963	0.996
Non-drinking									
Heterozygote	AG versus AA	0.69 (0.48–1.00)	0.048	0.956	0.136^b^	0.320	0.838	0.981	0.998
Dominant	GG + AG versus AA	0.69 (0.49–0.98)	0.040	0.964	0.106^b^	0.263	0.797	0.975	0.997

*OR* odds ratio, *CI* confidence interval

^a^*p* < 0.05 indicates statistical significance

^b^The level of false positive report probability threshold was set at 0.2 and noteworthy findings are presented

### GO and KEGG analysis

**Fig. 1:**
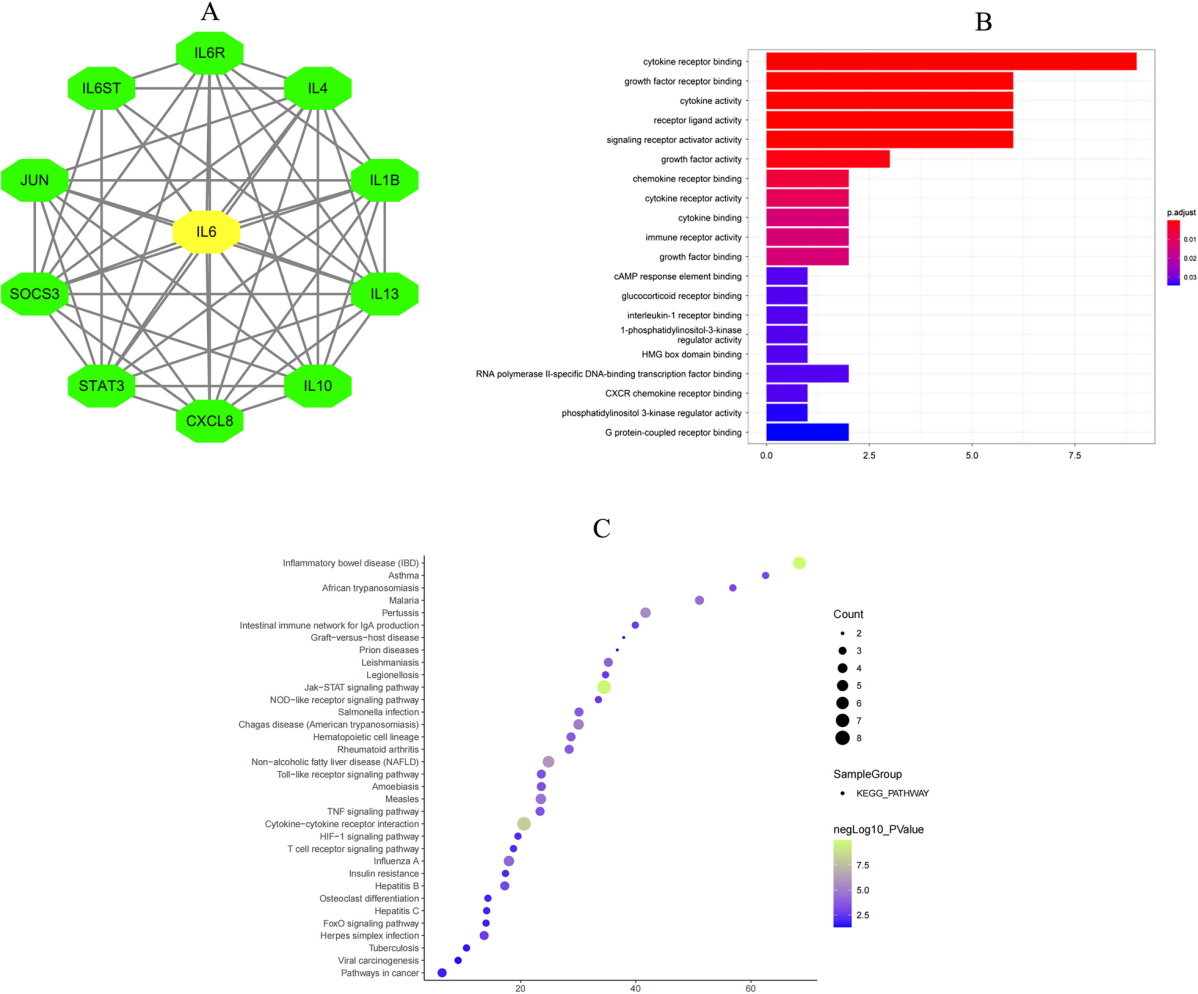
The potential function of *IL-6*. **A** Protein–protein interaction network of *IL-6*; **B** GO analysis of *IL-6*; **C** KEGG pathway analysis of *IL-6*

Using String database and Cytoscape software, a protein–protein interaction network for *IL-6* gene (Fig. 1A). The GO enrichment analysis showed that *IL-6* was mainly enriched in cytokine receptor binding and growth factor receptor binding (Fig. 1B). The results of KEGG pathway analysis revealed that *IL-6* is mainly involved in the Jak-STAT signaling pathway and rheumatoid arthritis (Fig. 1C).

## Discussion

In this case–control study, *IL-6* rs2069837 and rs13306435 were genotyped to investigate the susceptibility to ONFH risk in the Chinese Han population. We only found that rs2069837 in the *IL-6* gene was correlated with a decreased susceptibility to ONFH in the Chinese population. Some studies reported that age, gender, smoking and drinking exert the crucial role in the etiology of ONFH [3, 23]. Given that age, gender, smoking and drinking are the risk factor for ONFH, stratification analysis by sex (males and females), age (> 51 years and ≤ 51 years), smoking (yes and no) and drinking (yes and no) were performed to estimate the effect of these factor on the association between these variants and ONFH risk. The stratified results showed that *IL-6* rs2069837 reduced the risk of ONFH among males, nonsmokers, nondrinkers, and individuals with age > 51 years. These data demonstrated that rs2069837 G>A polymorphism might have a beneficial effect on the development of ONFH.

The *IL-6* gene is located on chromosome 7p15.3, containing six exons and five introns [24]. *IL-6* encodes a cytokine protein, which functions in inflammation, maintains immune homeostasis and plays an important role in bone metabolism [25]. Xie et al. have reported that IL-6 level increased during osteogenic differentiation in bone marrow-derived mesenchymal stem cells (BM-MSCs) and was positively correlated with the osteogenic potential of BM-MSCs [26]. A previous study showed that significant upregulation of IL-6 levels was observed in osteoporotic BMMCs compared with normal controls, suggesting IL-6 as a promising target for osteoporosis therapy [27]. And another study has indicated that IL-6 classic signaling is essential for the bone healing process [28]. These lines of evidence have demonstrated that *IL-6* gene played a crucial role in bone-related disease.

The rs2069837 polymorphism, located in the intron of *IL-6* gene, was identified as an important susceptibility variant of many diseases. Previous study has demonstrated that rs2069837 elevated papillary thyroid cancer risk among Chinese [29]. Some research also found a significant association of rs2069837 with an increased risk of cervical cancer in Eastern Chinese women [30, 31]. Renauer et al. have indicated that rs2069837 was correlated with increased risk of Takayasu’s arteritis in Turkey and North America people [32]. In addition, Chen et al. found that rs2069837 variant increased the susceptibility to rheumatoid arthritis among young people and males [21]. However, we found that rs2069837 is associated with a lower risk of ONFH among the Chinese Hans. The reason for these inconsistent results may be associated with factors such as type of disease, region, ethnicity, and sample size. Furthermore, rs2069837 might be associated with the regulation of promoter histone marks, enhancer histone marks, DNAse, proteins bound and motifs changed, suggesting its potential function in ONFH. Rs2069837 in *IL-6* might increase disease susceptibility by suppression of the anti-inflammatory gene GPNMB, but a direct effect from rs2069837 on *IL-6* expression was not detect [33]. The specific mechanisms of rs2069837 on ONFH occurrence require further investigation.

Rs13306435 was located in exon 5 of *IL-6*gene. The T>A variation of rs13306435 changed an amino acid from Asp to Glu. The T allele of rs13306435 had been reported previously to be associated with increased expression and plasma levels of *IL-6* [34]. Reportedly, Americans present the highest allele frequency of *IL6* rs13306435 (A = 0.078) among all ethnic groups (Global A = 0.020, the 1000 Genomes Project, Phase 3) [35]. *IL-6* rs13306435 was associated with hematological toxicity in leukemic patients [36], baseline peritoneal transport property [37]. However, no associations between rs13306435 and ONFH susceptibility were observed in the overall analysis and stratified analysis. The contribution of rs13306435 to ONFH risk need to further investigate in larger-scale prospective studies.

Although interesting results were found between rs2069837 variant and ONFH susceptibility, there were some limitations in this study. First of all, only two polymorphisms in *IL-6* gene were studied, more polymorphisms are needed to investigate. Second, all participants were Han Chinese, so we need more different ethnic populations to confirm our findings. Third, *IL-6* levels in plasma/serum or synovial fluid of ONFH patients and the association of *IL-6* SNPs and the mNRA expression were not be detected. In further studies, multiple SNPs in *IL-6* should be selected and genotyped to investigate the contribution of *IL-6* polymorphisms to ONFH risk, and further studies for functional effect of SNPs on *IL-6* expression are desired.

## Conclusions

To sum up, we suggested that rs2069837 G>A polymorphism in *IL-6* gene was significantly associated with a decreased risk of ONFH among the Chinese Hans. These findings underscored the crucial role of *IL-6* rs2069837 in the occurrence of ONFH.

## Supplementary Information

**Additional file 1:** Supplemental tables.

## Data Availability

The datasets generated during the current study are available in the [zenodo] repository, accession number: 5372106 (https://zenodo.org/record/5372106#.YXtJjnbE9N8).
