# Supplementary material for: An intron SNP rs2069837 in IL-6 is associated with osteonecrosis of the femoral head development
Source: BMC Med Genomics. 2022 Jan 5;15:5. doi: 10.1186/s12920-021-01142-3 (PMC8734317; doi:10.1186/s12920-021-01142-3)
Supplement: Supplementary file 1 — Additional file 1: Supplemental tables. [file 12920_2021_1142_MOESM1_ESM.docx]

Supplemental table 1 Primer sequence of rs2069837 and rs13306435 in *IL-6*

| SNP | 1^st^-PCRP | 2^nd^-PCRP | UEP_SEQ |
| --- | --- | --- | --- |
| rs2069837 | ACGTTGGATGCTGCTGGAACATTCTATGGC | ACGTTGGATGTCTCCAAAAACCTTCCTTGC | TTTTGAAGATTAGACACAATATTTAT |
| rs13306435 | ACGTTGGATGCCCTCCACTGCAAAGGATTT | ACGTTGGATGTGGCATTTGTGGTTGGGTCA | TCAGGGGTGGTTATTGC |

Supplemental table 2 Associations between *IL-6* polymorphisms and ONFH susceptibility stratified by stage

| SNP | Model | Genotype | I/II | III/IV | OR (95% CI) | *P* |
| --- | --- | --- | --- | --- | --- | --- |
| rs2069837 | Allele | A | 121 (78.6%) | 381 (85.4%) | 1.00 |  |
|  |  | G | 33 (21.4%) | 65 (14.6%) | 0.63(0.39-1.00) | 0.057 |
|  | Codominant | AA | 52 (67.5%) | 156 (70%) | 1.00 |  |
|  |  | GG | 1 (1.3%) | 5 (2.2%) | 0.56(0.31-1.02) | 0.057 |
|  |  | AG | 24 (31.2%) | 62 (27.8%) | 0.61(0.10-3.95) | 0.606 |
|  | Dominant | AA | 52 (67.5%) | 156 (70%) | 1.00 |  |
|  |  | GG+AG | 25 (32.5%) | 67 (30%) | 0.57(0.32-1.01) | 0.054 |
|  | Recessive | AA+AG | 76 (98.7%) | 218 (97.8%) | 1.00 |  |
|  |  | GG | 1 (1.3%) | 5 (2.2%) | 0.74(0.12-4.07) | 0.748 |
|  | Additive | / |  |  | 0.62(0.37-1.04) | 0.068 |
| rs13306435 | Allele | T | 148 (96.1%) | 429 (96.2%) | 1.00 |  |
|  |  | A | 6 (3.9%) | 17 (3.8%) | 0.98(0.38-2.53) | 0.963 |
|  | Codominant | TT | 71 (92.2%) | 206 (92.4%) | 1.00 |  |
|  |  | AA | 0 (0.0%) | 0 (0.0%) | / | / |
|  |  | AT | 6 (7.8%) | 17 (7.6%) | 1.36(0.47-3.95) | 0.560 |
|  | Dominant | TT | 71 (92.2%) | 206 (92.4%) | 1.00 |  |
|  |  | AA+AT | 6 (7.8%) | 17 (7.6%) | 0.77(0.28-2.13) | 0.615 |
|  | Recessive | TT+AT | 77 (100.0%) | 223 (100.0%) | 1.00 |  |
|  |  | AA | 0 (0.0%) | 0 (0.0%) | / | / |
|  | Additive | / |  |  | 0.77(0.28-2.13) | 0.615 |

SNP: single nucleotide polymorphism; OR: odds ratio; CI: confidence interval.

*P* values were calculated by logistic regression analysis adjusted age and sex.
